# The Effect on Extubation of Early vs. Late Definitive Closure of the Patent Ductus Arteriosus in Premature Infants: A Target Trial Emulation Using Electronic Health Records

**DOI:** 10.3390/jcm14062072

**Published:** 2025-03-18

**Authors:** Zhou Du, Craig R. Wheeler, Michael Farias, Diego Porras, Philip T. Levy, Arin L. Madenci

**Affiliations:** 1 Harvard Medical School, Boston, MA 02115, USA; zhou_du2@hms.harvard.edu (Z.D.); michael.farias@cardio.chboston.org (M.F.); diego.porras@cardio.chboston.org (D.P.); arin_madenci@g.harvard.edu (A.L.M.); 2 Division of Newborn Medicine, Boston Children’s Hospital, Boston, MA 02115, USA; craig.wheeler@childrens.harvard.edu; 3 Departments of Respiratory Care & Extracorporeal Membrane Oxygenation, Boston Children’s Hospital, Boston, MA 02115, USA; 4 Department of Cardiology, Boston Children’s Hospital, Boston, MA 02115, USA; 5 Department of Surgery, Boston Children’s Hospital, Boston, MA 02115, USA; 6 Department of Epidemiology, Harvard T. H. Chan School of Public Health, Boston, MA 02115, USA

**Keywords:** prematurity, patent ductus arteriosus, bronchopulmonary dysplasia, extubation success, target trial emulation, device closure, surgical ligation

## Abstract

**Background/Objectives**: Premature infants are often referred for the definitive procedural closure of the patent ductus arteriosus (PDA) with the failure of, or contraindication to, pharmacotherapy and the inability to wean respiratory support. However, once this need is identified, the importance of expedited closure is unclear. The objective of this study was to compare the effect of the timing of definitive closure (i.e., surgical ligation or device occlusion) on early respiratory outcomes in premature infants. **Method**: We first specify a hypothetical randomized trial (the “target trial”) that would estimate the effect on extubation of early (0–4 days from referral) vs. late (5–14 days from referral) definitive PDA closure. We then emulate this target trial using a single-institution registry of premature infants (born <30 weeks or with a birth weight < 1500 g) who underwent the definitive closure of PDA between January 2014 and October 2023. **Results**: We identify 131 eligible infants. At the end of the follow-up, 70 and 38 infants were adherent to early and late PDA closure strategies, respectively. The cumulative incidence of extubation in the early group was higher than that in the late group until day 40 (maximum risk difference: 22 percentage points at day 13; 95% CI: −11 to 56). Outcomes were similar at the end of the 45-day follow-up period (risk difference: −1 percentage point; 95% CI: −46 to 42). **Conclusions**: The need for mechanical ventilation was equivalent between early and late PDA closure strategies at the end of a 45-day follow-up period although infants in the early intervention group were extubated sooner.

## 1. Introduction

Premature infants with patent ductus arteriosus (PDA) causing cardiovascular compromise are often referred for definitive procedural closure when expectant management or pharmacotherapy fails, there is a contraindication to medications, or there remains an inability to wean mechanical ventilation. The decision to perform a definitive procedure via the surgical ligation or device occlusion of PDA is then balanced between the potential benefit of instantaneous closure and clinical improvement with the possibilities of peri-procedural instability and spontaneous improvement [1,2,3,4,5].

As the field continues to debate the need for PDA closure with uncertainty persisting around the “if” and “how” to treat, it remains critical to understand the impact that timing from referral (once the decision has been made to remove PDA with a definitive option) to the actual closure of PDA has on respiratory morbidity and other outcomes. PDA trials have often utilized short- and long-term respiratory morbidity as outcome measures because of the known associations of prolonged exposure of shunt volume with longer durations of mechanical ventilation and the development of bronchopulmonary dysplasia (BPD) [6,7,8,9]. Several studies have previously reported on the optimal timing of definitive closure [1,2,4,10,11,12]. However, important components of study design (such as heterogeneous eligibility criteria and vaguely defined interventions) and analysis (such as the unintentional creation of immortal time bias) deviate from the way in which a randomized control trial (RCT) would be implemented, limiting the interpretability of effect estimates. In addition, most studies examining the timing of definitive closure have focused on surgical ligation [1,2,4,10,11,12] while transcatheter device occlusion is now the prevailing approach for the definitive closure of PDA in premature infants [13,14].

Accordingly, the primary objective of this study was to compare the effect of time from referral to definitive closure on short-term respiratory outcomes in premature infants without complex congenital cardiac disease.

## 2. Materials and Methods

We first specify the hypothetical randomized trial (the “target trial”) we would want to perform to estimate the effect on extubation of early vs. late definitive PDA closure. We then emulate that trial using data from electronic health records. This two-step approach (i.e., first specifying and then emulating a target trial) mitigates common avoidable analytical errors in observational analyses.

### 2.1. Specification of the Target Trial

We describe a hypothetical randomized control trial enrolling premature infants at a single institution who were referred for definitive closure of PDA between January 2014 and October 2023 (Table 1).

#### 2.1.1. Eligibility Criteria

Infants would be eligible to participate in this study if they were born before 30 weeks of gestational age or had birth weights of less than 1500 g. All eligible participants would have PDA considered to be hemodynamically significant by a treating clinician and would be mechanically ventilated at the time of referral. Eligible infants would have received at least one course of pharmacotherapy prior to referral unless contraindicated. Neonates would be excluded in the presence of complex congenital cardiac disease (defined as anomalies other than hemodynamically insignificant atrial septal defect, ventricular septal defect, or patent foramen ovale), bidirectional or right-to-left direction of PDA flow, major congenital anomalies, or a tracheostomy.

#### 2.1.2. Treatment Strategies

Eligible participants would be assigned to undergo PDA closure within 0–4 days of randomization (“early” intervention) or between 5 and 14 days after randomization (“late” intervention). For either strategy, the determination of timing of intervention would be left to the treating physician’s discretion and logistical factors (e.g., surgeon or interventionalist availability, transport requirements, distance from high-volume center, healthcare insurance clearance, etc.). We refer to the number of days after which the specified intervention strategy may take place as a “grace period”.

#### 2.1.3. Treatment Assignment

Treatment would be assigned randomly in a non-blinded fashion.

#### 2.1.4. Outcome

The primary outcome of interest is successful extubation, defined as lack of invasive mechanical ventilation for at least seven days after extubation [15].

#### 2.1.5. Follow-Up

Beginning at the time of referral (time zero and randomization), the primary outcome would be assessed over a 45-day follow-up period. The time of referral would coincide with the times of eligibility assessment and treatment assignment.

#### 2.1.6. Casual Contrast

In the target trial, it would be straightforward to estimate the intent-to-treat effect or the effect of assignment to early or late intervention (regardless of adherence to this assignment). However, we would also estimate a non-naive per-protocol effect (specifically, the effect of adhering to the assigned treatment with complete follow-up), which may be of greater clinical interest.

#### 2.1.7. Analysis

To estimate this per-protocol effect using data from the target trial, we would fit a treatment model over the grace period for each intervention among all eligible individuals who had not deviated from their assigned treatment arm. This model could be adjusted for baseline and possibly post-baseline prognostic factors that predicted adherence. In this case, the adjustment set would include baseline covariates of birth weight, gestational age, age at referral, sex, year of enrollment, and prior attempt(s) of pharmacological treatments for PDA (Table S1). Of note, neonates who die prior to the study endpoint would be considered to be intubated until the end of the follow-up period, corresponding to a total effect type of estimand. (Estimation of an alternative estimand, the controlled direct effect, is described below in a sensitivity analysis.) We would then estimate inverse probability of treatment weights from this model and, subsequently, fit a weighted pooled logistic regression model to estimate the cumulative incidence of extubation over the follow-up period.

We could additionally perform three sensitivity analyses. First, the controlled direct effect (as compared with the total effect of the main analysis) could be estimated in the absence of competing risk events (e.g., had no participants died). Second, the main analysis could be repeated with the inclusion of referral site in the confounder set. Third, the estimated cumulative incidence of extubation could be compared between a strategy of intervention for PDA at a younger age (e.g., PDA closure between 15 and 20 days) and one at an older age (e.g., PDA closure between 21 and 35 days) among neonates referred for intervention before 14 days of age.

### 2.2. Emulation of the Target Trial

This study was approved by the Boston Children’s Hospital Institutional Review Board (IRB-P00035857). In 2019, we established a quality-improvement-based registry of all infants referred to Boston Children’s Hospital for consideration of definitive closure. We analyzed data from infants with PDA referred to a quaternary care center for surgical ligation or transcatheter closure. Infants’ demographic, clinical, echocardiographic, and procedural data had been entered into the registry. The date of referral for consideration of definitive closure had also been documented.

The eligibility criteria, treatment strategies, follow-up, and outcome were the same as those specified in the target trial above. We note the following differences in the target trial emulation.

#### 2.2.1. Treatment Assignment

Each eligible participant is classified into both strategies at baseline.

#### 2.2.2. Causal Contrast

The casual contrast is an observational analog of the per-protocol effect.

#### 2.2.3. Analysis

The analysis of the per-protocol effect would follow that of the target trial with the following exception. In the observed data, baseline labels of treatment assignment do not exist, and an individual’s data may be compatible with both treatment arms at a given time. That is, during the early grace period (days 0–4), a participant’s observed data may, at times, be consistent with both strategies. One analytical option would be to flip a fair coin for each individual and thereby randomly assign the treatment arms. Instead, for statistical efficiency, we create two clones of each individual and assign one clone to each of the two treatment strategies. Each eligible patient contributes one clone to each treatment arm so long as they remain compatible with that strategy. A clone would then be censored for deviation from the assigned treatment strategy at the time of deviation. The code for the analysis (R version 4.4.2) is available on GitHub (https://github.com/zhou996996/PDA-timing-study/tree/main (accessed on 11 March 2025)).

## 3. Results

**Figure 1:**
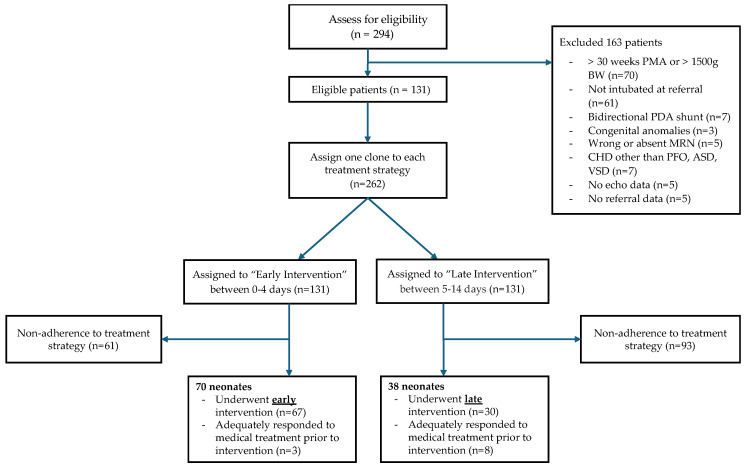
Study flowchart.

In the emulation of the target trial, a total of 131 neonates met the eligibility criteria between 1 January 2014 and 31 October 2023. At the end of the follow-up, 70 and 38 neonates had histories consistent with the early and late PDA closure strategies, respectively. One neonate followed both strategies due to early extubation without intervention; 24 neonates initially followed one or both strategies but ultimately deviated from both strategies. Figure 1 displays a flowchart of the study population.

The median gestational ages (24.5 weeks vs. 24.5 weeks) and birth weights (745 g vs. 732.5 g) were similar in the early and late groups. Over 90% of neonates had PDA only for both groups (Table 2). After adjustment, characteristics were generally similar between the groups, except that neonates in the early intervention group were more frequently white (52% vs. 41%) compared with those in the late intervention group. During the last assessment before PDA intervention, the diameters of the duct (2.5 mm vs. 2.4 mm) were also similar in the early and the late groups (Table S2).

**Figure 2:**
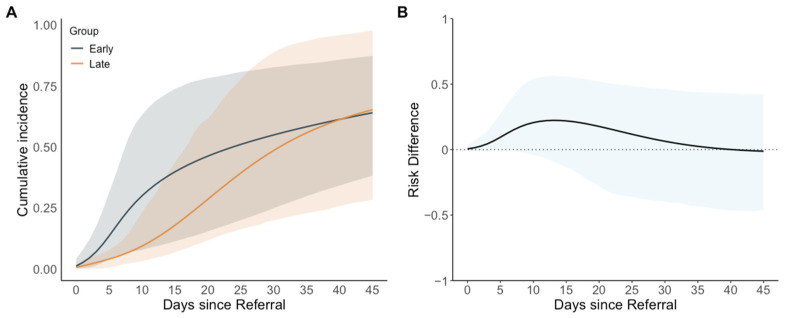
Estimated cumulative incidence and cumulative incidence differences in extubation since referral for patent ductus arteriosus intervention, comparing early (0–4 days from referral) with late (5–14 days from referral) interventions. (**A**) Estimated cumulative incidence (95% confidence interval shaded); (**B**) estimated cumulative incidence difference (95% confidence interval shaded) using the late group as the reference.

The estimated cumulative incidence of extubation at day 14 from referral was higher in the early group (38%; 95% CI: 10 to 72%) than in the late group (16%; 95% CI: 6 to 39%) with a cumulative incidence difference of 22 percentage points (95% CI: −11 to 56). At the end of the 45-day follow-up period, the estimated cumulative incidence values of extubation were similar between the groups (64%; 95% CI: 38 to 87% in the early group and 65%; 95% CI: 28 to 98% in the late group) with a cumulative incidence difference of −1 percentage point (95% CI: −46 to 42). Neonates in the early intervention group were extubated sooner than those in the late intervention group until day 35. The maximum cumulative incidence difference occurred at day 13 (21 percentage points, 95% CI: −11 to 55) with cumulative incidence values of extubation of 36% (95% CI: 10 to 71%) in the early group and 15% (95% CI: 5 to 38%) in the late group. The cumulative incidence of extubation over the 45-day follow-up period and cumulative incidence differences are illustrated in Figure 2 and Table 3. The estimated mean ventilator days were lower in the early group than in the late group throughout the study. At the end of follow-up period, they were 26.3 days (95% CI: 14.8 to 37.4) and 30.8 days (95% CI: 19.3 to 39.4) in the early and late group, respectively, with a difference of −4.5 days (95% CI: −18.5 to 11.5). 

**Figure 3:**
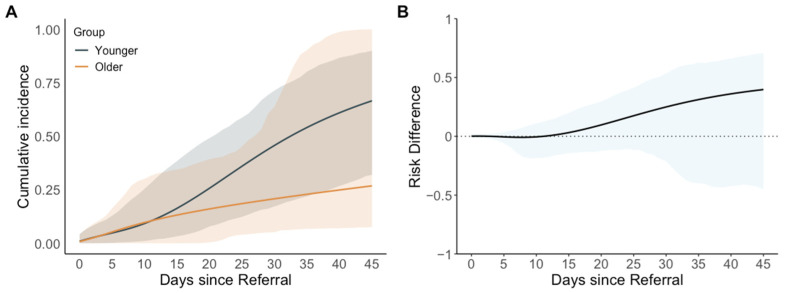
Estimated cumulative incidence and cumulative incidence difference comparing strategies of patent ductus arteriosus closure at a younger (15 to 20 days of life at intervention) vs. older age (21 to 35 days of life at intervention) for neonates referred before 14 days of age (sensitivity analysis #3). (**A**) Estimated cumulative incidence (95% confidence interval shaded); (**B**) estimated cumulative incidence difference (95% confidence interval shaded), using the older group as the reference.

Events and outcomes at the end of the grace period are summarized in Table S3. The results favored earlier repair in a sensitivity analysis comparing intervention at a younger age (15 to 20 days) with intervention at an older age (21 to 35 days) among neonates referred for intervention before 14 days of age (Figure 3 and Table 4).

The results of the sensitivity analyses 1 and 2 are similar to those of the main analysis. Neonates in the early intervention group were extubated sooner than those in the late intervention group for most of the study period while the cumulative incidence was similar between groups at the end of the follow-up period. The results of sensitivity analysis 1 are presented in Figure S1 and Table S4. Those of sensitivity analysis 2 are presented in Figure S2 and Table S4.

## 4. Discussion

Our findings suggest that premature infants without complex congenital heart disease who undergo earlier definitive PDA closure extubate sooner compared with those who undergo a delayed intervention. Over a longer-term (45-day) follow-up, there was no difference in the extubation rates based on timing from referral to definitive closure, but those in the early closure group were exposed to fewer ventilator days. As such, these results support an urgency for the definitive closure of PDA.

Prior studies evaluating the timing of definitive PDA closure have deviated from study design principles in a number of ways that may introduce bias. First, the analyses have generally made oversimplified comparisons of outcomes based on the ages of neonates who underwent PDA closure [1,2,3,4,10,11]. In doing so, eligibility criteria have been applied to the groups at different times, which may have introduced immortal time bias. For example, individuals in a late group must, implicitly, have survived and remained intubated, with symptomatic PDA, to be included in that group. Additionally, defining the treatment strategy using only age (e.g., <14 or <21 days after birth) without establishing a time of baseline (when eligibility would be assessed and follow-up would begin) does not consider when the PDA became symptomatic or the time of first eligibility. By introducing a grace period and including participants in as many treatment strategies as they are eligible for, the present study describes how to avoid this type of bias. When we applied this methodology to a strategy of PDA closure based on age in a sensitivity analysis, estimates were imprecise but favored a strategy of PDA closure at a younger age.

The optimal time to close hemodynamically significant PDA (hsPDA) has previously been explored in the context of “length of exposure” under the assumption that PDA causes cardiovascular compromise from birth until the date of closure [16,17,18]. There is evidence suggesting that prolonged exposure to hsPDA impacts both short- and long-term respiratory morbidity [7,18], which is even more pronounced among neonates who remain mechanically ventilated for greater than 10 days [19]. The timing from referral to definitive closure (a surrogate for the identification of the hemodynamic significance) may better reflect the actual length of exposure. Additionally, the results of these prior studies are difficult to interpret given that length of exposure is not in and of itself a well-defined clinical intervention (unlike a procedure to close PDA).

The impact of the timing of definitive PDA closure on clinical outcomes has generally been explored in studies of open surgical PDA ligation via thoracotomy. Until recently, the primary method for definitive closure of PDA was surgical ligation in premature infants < 2 kg. Following the United States Food and Drug Administration approval of a transcatheter occlusive device in premature infants in 2019, our practice changed to offering this minimally invasive approach as a first-line option. The rapid adoption of transcatheter device occlusion as the primary definitive closure option in premature infants has pushed the field to consider its impact on outcomes separate from surgical ligation [14]. Earlier referral and subsequent prompt closure for premature infants who need their PDA closed may improve short-term respiratory outcomes. In our study, the 30 and 45-day cumulative incidence of extubation for infants referred for intervention before 14 days of life was decreased for those undergoing closure at a younger age (at 15 to 20 days of life, compared with 21 to 35 days of life).

Given the clinical importance of mitigating the consequences of PDA that may cause cardiopulmonary harm in premature infants, advances in how we define the timing for exposure may lead to more reliable detection and serial assessments of disease burden and inform decision-making processes. Variability remains on how a pathological PDA is defined and treated when the volume of the shunt is sufficiently large that cardiovascular compromise may ensue, especially for infants who have been mechanically ventilated for a prolonged period. As such, reducing the total number of ventilator days by expeditious PDA closure may ultimately improve both short- and long-term outcomes.

There were several limitations to this study. Intervention timing was not assigned randomly and the potential for unmeasured confounding exists, especially without the ability to adjust for possible post-baseline confounders. The decision for the neonates receiving late intervention was based on the treating physician’s discretion and logistical restraints. However, the specific reasons were unavailable to the researchers. Furthermore, there may be situations in which the decision to intervene against PDA is not captured in the available data or based on clinician’s bedside discretion in such a way that lacks equipoise. We tried to limit this occurrence by implementing relatively strict eligibility criteria and we attempted to emulate the randomization of treatment strategy by including baseline covariates of birth weight, gestational age, age at referral, sex, year of referral, and whether the patients had had pharmacological treatments for PDA at referral in the treatment model. However, we did not have access to information on several potentially relevant baseline and post-baseline confounders (i.e., echocardiogram and ventilator settings at referral, BPD, and associated pulmonary hypertension). Loss to follow-up due to retro-transfer after intervention was frequent and may have introduced bias. Finally, the small sample size precluded sensitivity analysis stratified by the number of PDA medical treatment courses, closure type (thoracotomy vs. transcatheter), or referral year.

## 5. Conclusions

In summary, we specified and then emulated a target trial of timing of PDA closure using data from electronic health records. This analysis suggests that early PDA closure strategies following referral for a definitive procedure may lead to short-term respiratory benefit among premature infants with symptomatic PDA. Future studies could focus on examining long-term outcomes such as the risk of BPD or mortality. This would provide a more comprehensive understanding of the effect of the timing of the definitive procedural closure of PDA.

## Figures and Tables

**Table 1:** Specification of the target trial and description of its emulation.

Protocol Component	Specification of the Target Trial	Target Trial Emulation
Eligibility criteria	Inclusion criteria: - Born <30 weeks gestational age or birth weight less than 1500 g - Mechanically ventilated at referral - Have a diagnosis of PDA confirmed by echocardiography and referred for closure intervention - Failed, or have contraindications to, at least one pharmacologic therapy * Exclusion criteria: - Complex congenital cardiac disease - Bidirectional or R-L flow in PDA - Major congenital anomalies	Same as target trial
Treatment strategies **	(1) Undergo “early” intervention for PDA closure within 0–4 days of randomization (2) Undergo “late” intervention for PDA closure within 5–14 days of randomization	(1) Undergo “early” intervention for PDA closure within 0–4 days of referral (2) Undergo “late” intervention for PDA closure within 5–14 days of referral
Treatment assignment	Random assignment to a treatment arm without blinding	Each individual is classified into both strategies at baseline
Primary outcome	Extubation	Same as target trial
Follow-up period	Follow-up begins at the time of assignment and ends at 45 days or at death, whichever occurs first	Same as target trial
Causal contrast	Per-protocol effect	Observational analog of per-protocol effect
Analysis plan	Per-protocol analysis: censoring individuals if/when they do not adhere to their treatment assignment, with inverse probability of treatment weighting to adjust for selection bias	Same as target trial with the following modification: Since treatment assignment is unknown, eligible individuals contribute clones to each treatment arm. A given clone is censored at the time of deviation from the assigned treatment strategy

* Including indomethacin, ibuprofen, and/or acetaminophen. ** In the target trial specification, the specific day of intervention within the assigned range (grace period) is left to the treating physician’s discretion and logistical factors; in the target trial emulation, the analytical choice is made to specify that neonates undergo interventions in a uniform distribution within the assigned range.

**Table 2:** Characteristics of eligible individuals referred for intervention for PDA closure (2014–2023) at the end of the grace period.

	Early Group *n* = 70 Unweighted	Late Group *n* = 38 Unweighted	Early Group *n* = 166 Weighted	Late Group *n* = 94 Weighted
GA at birth, wks	25 [24, 25]	25 [2, 24]	24 [23, 25]	24 [24, 25]
PMA at referral, wks	28 [27, 28]	27 [25, 28]	27 [25, 28]	27 [27, 28]
Age at referral, days	19 [13, 24]	13 [6, 21]	15 [11, 23]	20 [13, 26]
Birth weight, g	745 [633, 859]	733 [593, 870]	680 [550, 852]	736 [604, 849]
Female (%)	28 (40.0)	22 (57.9)	78 (46.7)	38 (40.3)
Year of referral	2018 [2015, 2020]	2021 [2016, 2022]	2019 [2015, 2022]	2017 [2015, 2020]
APGAR1 *	3.0 [1.0, 5.0]	3.0 [1.0, 6.0]	4.0 [2.0, 5.0]	3.0 [1.0, 5.0]
APGAR5 *	6.5 [5.0, 8.0]	6.0 [5.5, 8.0]	7.0 [5.0, 7.0]	6.0 [5.0, 8.0]
Race (%)				
White	27 (38.6)	13 (34.2)	86 (51.9)	39 (41.4)
Black	16 (22.9)	12 (31.6)	28 (17.1)	20 (21.7)
Asian	2 (2.9)	3 (7.9)	3 (1.9)	4 (4.0)
Other/Unknown	25 (35.7)	10(26.3)	48 (29.1)	31 (32.0)
Surfactant use (%)	67 (95.7)	33 (86.8)	161 (96.8)	88 (93.8)
Inotropes use *	11 (19.0)	10 (43.5)	28 (26.3)	19 (24.3)
PDA medications **	61 (87.1)	28 (73.7)	133 (80.1)	81 (86.3)
CHD type				
Only PDA	65 (92.9)	36 (94.7)	159 (96)	88 (93.3)
PDA and ASD	4 (5.7)	1 (2.6)	5 (3)	5 (5)
PDA and VSD	1 (1.4)	1 (2.6)	2 (1)	2 (1.7)

Data are presented as median (interquartile range) or number (percent); ASD: atrial septal defect; CHD: congenital heart disease; GA: gestational age; PMA: postmenstrual age; PDA: patent ductus arteriosus; VSD: ventricular septal defect. * Missing data: APGAR (*n* = 5, 4%) and inotrope use (*n* = 34, 26%). ** PDA treatment courses were missing for 15 neonates (12%), who were counted as not having had PDA treatment.

**Table 3:** Estimated cumulative incidence of extubation and differences at selected time points.

	Day 7	Day 14	Day 30	Day 45
Early PDA intervention (%)	21 (6, 47)	38 (10, 72)	55 (25, 83)	64 (38, 87)
Late PDA intervention (%)	6 (2, 12)	16 (6, 39)	49 (20, 89)	65 (28, 98)
Difference (ref. late) (%)	15 (0, 41)	22 (−11, 56)	6 (−40, 46)	−1 (−46, 42)

PDA: patent ductus arteriosus.

**Table 4:** Estimated cumulative incidence of extubation and differences between a strategy of PDA closure at a younger (14 to 20 days of life at intervention) vs. one at an older age (21 to 35 days of life at intervention) at selected time points among neonates referred before 14 days of age (sensitivity analysis #3).

	Day 7	Day 14	Day 30	Day 45
Younger group (%)	6 (0, 18)	15 (3, 36)	46 (17, 71)	67 (32, 90)
Older group (%)	7 (0, 23)	13 (0, 34)	21 (5, 64)	27 (8, 100)
Difference (ref. older) (%)	−1 (−14, 6)	2 (−15, 18)	25 (−21, 53)	40 (−45, 71)

PDA: patent ductus arteriosus.

## Data Availability

All data have been presented in this study. Further inquiry can be directed to the corresponding author. The code used in this analysis is available online: https://github.com/zhou996996/PDA-timing-study/tree/main (accessed on 13 March 2025).

## Supplementary Materials

The following supporting information can be downloaded at: https://www.mdpi.com/article/10.3390/jcm14062072/s1, Table S1. Variables used in the analysis when emulating the target trial; Table S2. Summary of echocardiogram parameters from the last assessment before PDA intervention among eligible individuals who were referred for intervention for PDA closure, at the end of the grace period; Table S3. Summary of events and outcome among eligible individuals who were referred for intervention for PDA closure, at the end of the grace period; Table S4. Estimated cumulative incidence of extubation and difference at selected time points for sensitivity analyses 1 and 2; Figure S1. Estimated cumulative incidence and cumulative incidence difference of extubation since referral for patent ductus arteriosus intervention, comparing early (0–4 days from referral) with late (5–14 days from referral) interventions, with analysis treating death as a censoring event (sensitivity analysis #1); Figure S2. Estimated cumulative incidence and cumulative incidence difference of extubation since referral for patent ductus arteriosus intervention, comparing early (0–4 days from referral) with late (5–14 days from referral) interventions, with analysis including referral site in the confounder set (sensitivity analysis #2).
