# Supplementary material for: The Effect on Extubation of Early vs. Late Definitive Closure of the Patent Ductus Arteriosus in Premature Infants: A Target Trial Emulation Using Electronic Health Records
Source: J Clin Med. 2025 Mar 18;14(6):2072. doi: 10.3390/jcm14062072 (PMC11943036; doi:10.3390/jcm14062072)
Supplement: Supplementary file 1 [file jcm-14-02072-s001.zip › jcm-3485341-supplementary.pdf]

Supplementary Materials

Expanded Results

Tables / Figure	Page #
**Table S1**: Variables used in the analysis when emulating the target trial	2
**Table S2:** Summary of echocardiogram parameters from the last assessment before PDA intervention among eligible individuals who were referred for intervention for PDA closure, at the end of the grace period	2
**Table S3:** Summary of events and outcome among eligible individuals who were referred for intervention for PDA closure, at the end of the grace period	3
**Table S4.** Estimated cumulative incidence of extubation and difference at selected time points for sensitivity analyses 1 and 2.	3
**Figure S1**. Estimated cumulative incidence and cumulative incidence difference of extubation since referral for patent ductus arteriosus intervention, comparing early (0-4 days from referral) with late (5-14 days from referral) interventions, with analysis treating death as a censoring event (sensitivity analysis #1).	4
**Figure S2.** Estimated cumulative incidence and cumulative incidence difference of extubation since referral for patent ductus arteriosus intervention, comparing early (0-4 days from referral) with late (5-14 days from referral) interventions, with analysis including referral site in the confounder set (sensitivity analysis #2).	4

**Table S1**. Variables used in the analysis when emulating the target trial		
Variable	Functional form	Value
Gestational age (days)	Splines	Continuous
Age at referral (weeks)	Splines	Continuous
Female	Indicator	Binary
Birth weight (grams)	Splines	Continuous
Pharmacologic intervention for PDA	Indicator	Binary
Follow-up time (days)	Splines	Continuous
Year of referral	Splines	Continuous

Table S2. Summary of echocardiogram parameters from the last assessment before PDA intervention among eligible individuals who were referred for intervention for PDA closure, at the end of the grace period.

	Early group N=70 Unweighted	Late group N=38 Unweighted	Early group N=166 Weighted	Late group N=94 Weighted
Age at echocardiogram, days	21.0 [14.0, 27.0]	22.5 [14.5, 30.8]	19.0 [14.0, 26.0]	23.0 [16.0, 28.0]
Diameter of duct, mm*	2.5 [2.2, 3.0]	2.4 [2.0, 2.5]	2.4 [2.0, 2.9]	2.5 [2.0, 3.0]
Peak systolic velocity of shunt, mm Hg*	20.0 [15.0, 25.0]	20.0 [15.0, 35.0]	20.6 [15.0, 25.0]	21.9 [15.0, 25.0]
Retrograde diastolic flow in Doppler (%)*	45 (76.3)	14 (50.0)	85 (76.2)	56 (70.4)
Dilation of left atrium (%)*	50 (72.5)	24 (66.7)	96 (58.5)	65 (71.0)
Dilation of left ventricle (%)*	49 (72.1)	19 (52.8)	86 (52.3)	62 (68.3)

Data is presented as median (Interquartile range) or number (percentage); PDA, patent ductus arteriosus.

*Missing data: diameter of duct, peak systolic velocity of shunt, retrograde diastolic flow in Doppler, dilation of left atrium and ventricle are missing for 15 (11.5%), 57 (43.5%), 28 (21.4%), 4 (3.1%) and 5 (3.8%) neonates in the unweighted dataset.

Table S3. Summary of events and outcome among eligible individuals who were referred for intervention for PDA closure, at the end of the grace period.

	Early group<br>N=70<br>Unweighted	Late group<br>N=38<br>Unweighted
Transcatheter closure (%)	20 (28.6)	12 (31.6)
Intervention since referral, days	1.0 [1.0, 3.0]	8.0 [6.8, 10.0]
Mechanical ventilation since referral, days	6.0 [4.0, 33.8]	16.0 [10.5, 32.8]
Intervention day of age, days	21.0 [14.0, 27.0]	22.5 [14.5, 30.8]
Event (%)		
Extubation	23 (32.9)	24 (63.2)
Lost to follow-up*	33 (47.1)	9 (23.7)
Death	2 (2.9)	1 (2.6)
Administrative censoring	12 (17.1)	4 (10.5)

Data is presented as median (Interquartile range) or number (percentage).

*Lost to follow-up was due to retrotransfer.

Table S4. Estimated cumulative incidence of extubation and difference at selected time points for sensitivity analyses 1 and 2.

	Day 7	Day 14	Day 30	Day 45
**Sensitivity analysis 1***				
Early PDA intervention (%)	21 (6, 47)	38 (10, 72)	55 (22, 83)	64 (30, 87)
Late PDA intervention (%)	6 (2, 12)	16 (6, 39)	55 (18, 89)	77 (2, 98)
Difference (ref. late) (%)	15 (0, 42)	22 (-11, 56)	0 (-46, 46)	-12 (-52, 40)
**Sensitivity analysis 2****				
Early PDA intervention (%)	31 (5, 78)	52 (10, 94)	65 (23, 95)	72 (35, 96)
Late PDA intervention (%)	6 (0, 14)	15 (2, 60)	48 (6, 99)	66 (7, 100)
Difference (ref. late) (%)	25 (-1, 74)	36 (-18, 80)	18 (-44, 67)	6 (-47, 70)

PDA, patent ductus arteriosus.

* Sensitivity analysis after truncating the follow-up at time of death for 3 death cases.

** Sensitivity analysis after including referral site in the confounder set.

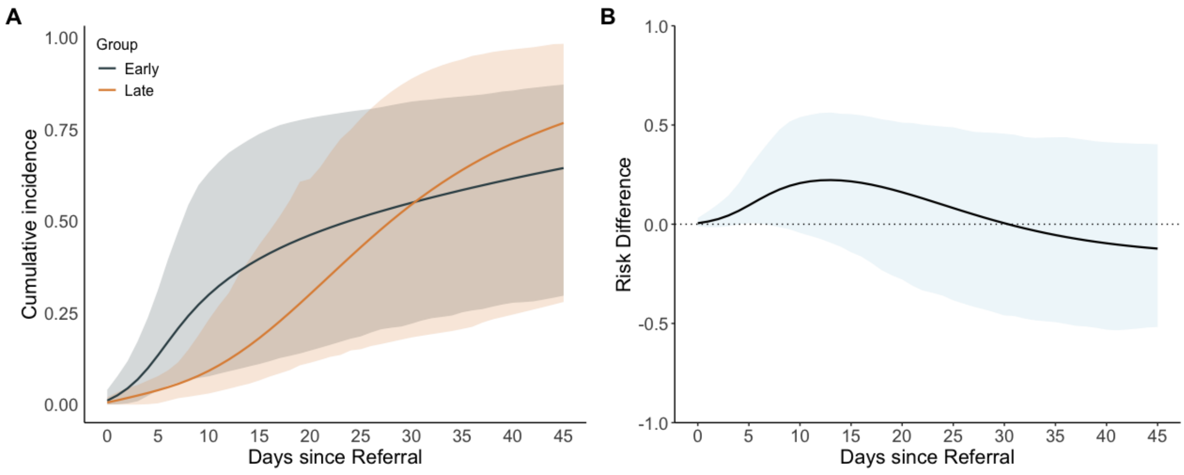

Figure S1. Estimated cumulative incidence and cumulative incidence difference of extubation since referral for patent ductus arteriosus intervention, comparing early (0-4 days from referral) with late (5-14 days from referral) interventions, with analysis treating death as a censoring event (sensitivity analysis #1).

(A) Estimated cumulative incidence (95% confidence interval shaded); (B) Estimated cumulative incidence difference (95% confidence interval shaded), using the late group as the reference.

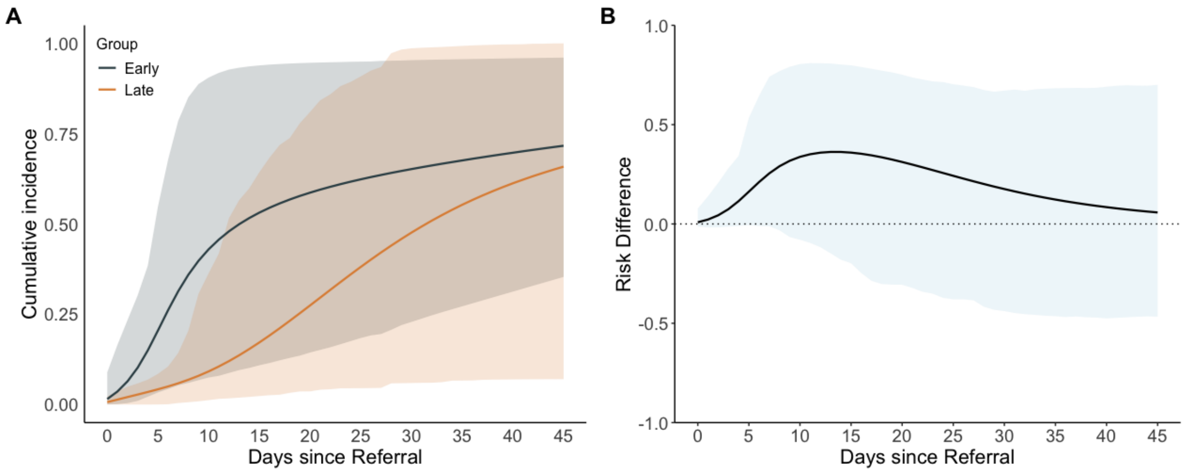

Figure S2. Estimated cumulative incidence and cumulative incidence difference of extubation since referral for patent ductus arteriosus intervention, comparing early (0-4 days from referral) with late (5-14 days from referral) interventions, with analysis including referral site in the confounder set (sensitivity analysis #2).

(A) Estimated cumulative incidence (95% confidence interval shaded); (B) Estimated cumulative incidence difference (95% confidence interval shaded), using the late group as the reference.
